# In vitro analysis of a novel dimethylaminododecyl methacrylate modification of dental acrylic soft liner material

**DOI:** 10.1038/s41598-024-69836-z

**Published:** 2024-08-27

**Authors:** Mohamed M. Ammar, Hala A. Elkammar, Abdelfattah A. Abdelkhalek, Nada A. Abdelrazek, Ahmed A. Emam, Bassem M. Abdelhameed

**Affiliations:** 1 Biomaterials Department, Faculty of Oral and Dental Medicine, Future University in Egypt, New Cairo, 11865 Egypt; 2 Oral Pathology Department, Faculty of Oral and Dental Medicine, Future University in Egypt, New Cairo, 11865 Egypt; 3 Microbiology Department of Supplementary General Science, Faculty of Oral and Dental Medicine, Future University in Egypt, New Cairo, 11865 Egypt; 4 Microbiology and Immunology Department, Faculty of Pharmacy, Future University in Egypt, New Cairo, 11865 Egypt; 5 Medical Experimental Research Center (MERC), Faculty of Medicine, Mansoura University, Mansoura, 35511 Egypt; 6 Removable Prosthodontic Department, Faculty of Oral and Dental Medicine, Future University in Egypt, New Cairo, 11865 Egypt

**Keywords:** Soft liner, Dimethylaminododecyl methacrylate, Biofilm, Cytotoxicity, Hardness, Health care, Materials science

## Abstract

Soft denture liners have limitations like short lifespan and increased microbial buildup. Despite promise as a non-leaching antimicrobial polymer in dentistry, the impact of dimethylaminododecyl methacrylate (DMADDM) on soft liner performance remains unexplored. This study aimed to evaluate the effect of integrating different concentrations of DMADDM to cold cure acrylic resin soft liner, on its antimicrobial activity, cytotoxicity, and physical properties. The same properties were compared to a conventional commercially available denture soft liner. The study employed a control group (conventional soft liner) and three test groups containing 3.3%, 6.6%, and 10% (total mass fraction) DMADDM, respectively. Antimicrobial activity against *Candida albicans* and *Streptococcus mutans* was assessed through colony counts and biofilm biomass. Cytotoxicity was evaluated using an oral epithelial cell line. Additionally, wettability and hardness were measured to assess physical properties. Incorporation of DMADDM significantly reduced *Candida albicans* and *Streptococcus mutans* counts, and biofilm biomass, compared to the control. Additionally, DMADDM improved the soft liner's wettability and mitigated long-term hardness increase. In conclusion, DMADDM holds promise in enhancing soft liner performance. However, careful selection of its optimum concentration is crucial to ensure both safety and efficacy for future clinical use.

## Introduction

Even the most meticulously crafted dentures require relining over time. This is because inevitable and irreversible resorption of the alveolar bone progressively alters the shape of the ridge^1^. Denture relining with soft liners offers a cost-effective and time-saving solution to address this issue. These liners typically last for several years and come in various types, providing cushioning, better pressure distribution, and enhanced comfort for patients with sensitive gums^2,3^. Soft liners are also beneficial after surgical procedures, for maxillofacial reconstructive prostheses, and for immediate dentures. Studies have shown that they can improve oral-health-related quality of life, masticatory function, and overall patient satisfaction^4^.

Dentures with acrylic-resin soft liners provide enhanced comfort but face limitations like color instability, shortened lifespan, and increased microbial growth due to porosity. To address these issues, researchers are developing more effective antimicrobial soft-liner materials. These materials aim to deliver lasting efficacy against microbes while preserving the mechanical properties necessary for proper denture function^5^. While many attempts have been made to modify soft liners with various antimicrobial agents, some limitations remain. Certain liners exhibited short-term effectiveness, while others reportedly compromised the mechanical integrity of the dentures^6–9^.

Quaternary ammonium methacrylates (QAMs) have been used in many resin structures as polymeric biocides for different applications^10^. Among these applications are dental ones as biocides in dental adhesives^11,12^, polymeric filling materials^6,13^, and polymeric prostheses^14,15^. The mechanism of action of QAMs as bacteriocidal polymers depends on their chemical structure which consists of a quaternary ammonium group that has a positive charge capable of causing electric imbalance in bacterial cell membrane upon contact, leading to cytoplasmic leakage and subsequent cell death^13^.

Dimethylaminododecyl methacrylate (DMADDM) is one of the QAMs that has been successfully used in denture base acrylic hard resin as a non-released contact-killing antibacterial polymer^14^ providing long-lasting antibacterial benefits. Several studies have evaluated its physical performance and antibacterial/antifungal properties^16,17^ which are essential in avoiding the risk of denture-related infections, particularly for immunocompromised individuals or those with compromised oral hygiene^6,14,18^.

However, like any promising newcomer, DMADDM comes with its own set of questions and challenges, especially regarding new applications like its incorporation in soft-liner material. When testing such a monomer at different concentrations to attain the best antimicrobial results, cytotoxicity and physical properties may be limiting factors. Determining the optimal DMADDM concentration for acrylic-based soft-liner and its cytotoxic effect on oral epithelial cells has never been reported in the literature, to date, to the best of our knowledge.

The present study aimed to evaluate the effect of incorporating different concentrations of DMADDM to cold-cure acrylic-resin soft-liner on its antimicrobial activity, cytotoxicity, and physical properties. The same properties of the conventional commercially available denture soft-liner were determined and compared with the new DMADDM-modified one. Thus, the null hypothesis to be tested was that the use of DMADDM in acrylic resin soft liner has no significant effect on its antimicrobial activity, cytotoxicity, and physical properties compared to control without DMADDM modification.

## Results

### Microbiological evaluation

#### Counts

**Figure 1:**
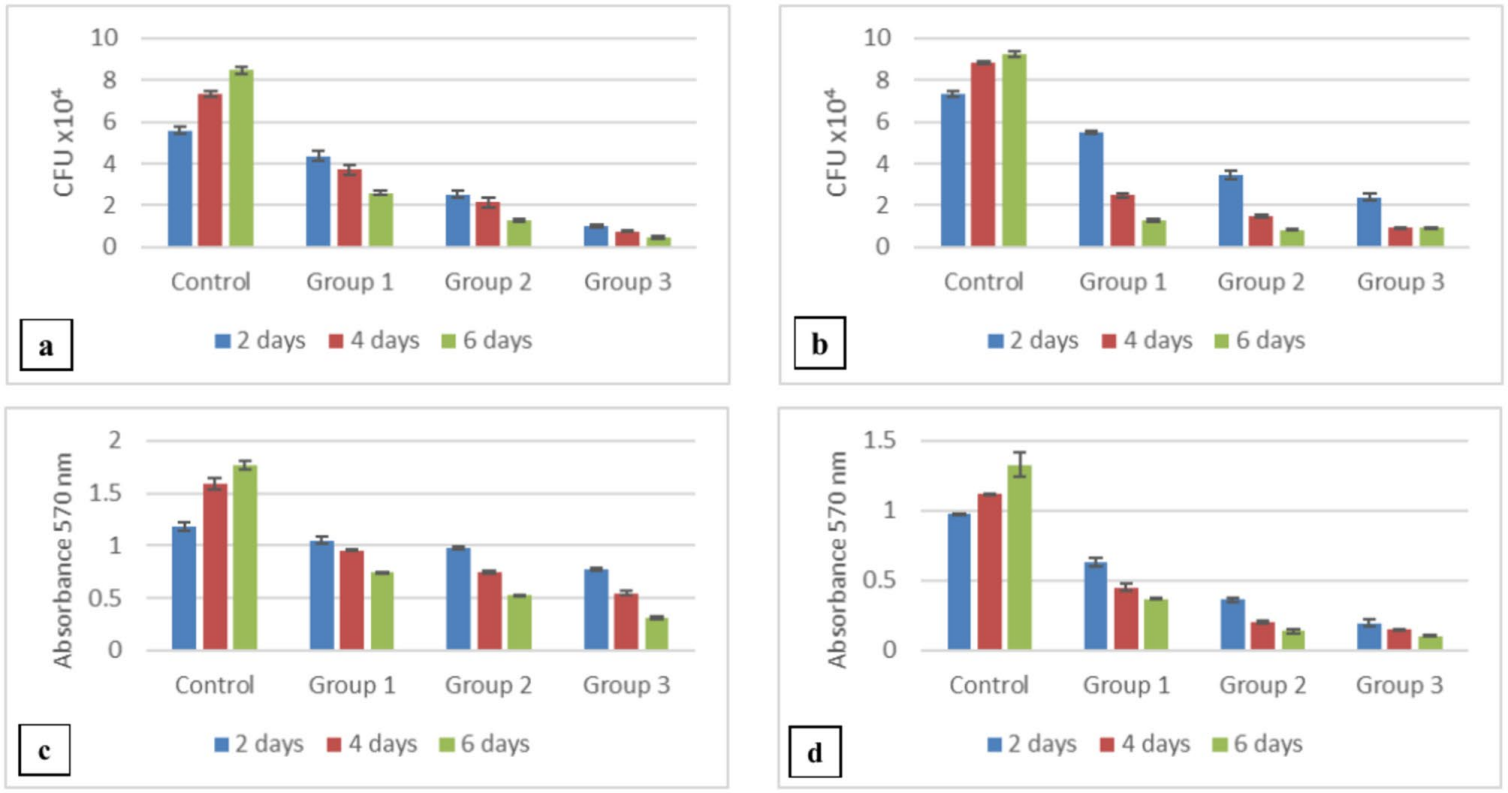
Microbiological evaluation results (means and standard deviations) of the study groups on days 2, 4, and 6: (**a**) *C. albicans* CFU count; (**b**) *S. mutans* CFU count; (**c**) *C. albicans* biofilm biomass; (**d**) *S. mutans* biofilm biomass. Post Hoc analysis showed a statistically significant difference between any two groups at p ≤ 0.05.

There was an increase in *Candida albicans* and *Streptococcus mutans* counts in the control group between day 2 (5.60 ± 0.20 and 7.33 ± 0.15, respectively) and day 6 (8.47 ± 0.15 and 9.23 ± 0.15, respectively) while all the test groups exhibited a decrease in counts between days 2 and 6. *C. albicans* counts mean values among the control group were higher than the other mean values of groups 1, 2, and 3. The lowest mean values of *C. albicans* count were reported among group 3 (0.46 ± 0.05) (Fig. 1a). On performing post hoc analyses, significant differences in *C. albicans* counts between any two groups were noted. Regarding *S. mutans* the highest counts were observed in the control group (9.23 ± 0.15) and the lowest counts were in group 2 (0.83 ± 0.01) (Fig. 1b). Post hoc test showed that there was a significant difference between any two groups.

When comparing the counts of the same group over different durations using post hoc tests, it was found that all groups showed a significant change for *C. albicans* (p = 0.005, 0.010, 0.009, and 0.002, for groups C, G1, G2, and G3 respectively between days 2 and 6) and for *S. mutans* (p = 0.004, 0.001, 0.002 and 0.006 for groups C, G1, G2 and G3 respectively between days 2 and 6). It was also noted that differences in *C. albicans* count between days 2 and 4 for groups 1 and 2 exhibited a non-significant difference (p-value = 0.104 and 0.184, respectively).

#### Biofilm biomass

The results revealed that for the control group, there was an increase in *C. albicans* and *S. mutans* biofilm biomass between day 2 (1.18 ± 0.04 and 0.98 ± 0.001, respectively) and day 6 (1.77 ± 0.04 and 1.33 ± 0.09, respectively) while all the test groups exhibited a decrease in biofilm biomass between days 2 and 6. The mean values of biofilm biomass of *C. albicans* were highest in the control group than in the other study groups on days 2, 4, and 6, while group 3 had the lowest values (0.31 ± 0.01) (Fig. 1c). A significant difference among groups was noted on using ANOVA test (p < 0.001). Post hoc analyses indicated significant differences between any two groups. Similar patterns were observed for the biofilm biomass of *S. mutans* (Fig. 1d). When comparing the biomass of the same group over different durations for *C. albicans* and *S. mutans* using post hoc tests, it was found that all groups showed a significant change between day 2 and day 6 (p values for *C. albicans* = 0.023, 0.005, 0.001 and 0.010 and p values for *S. mutans* = 0.004, 0.001, 0.002 and 0.006 for groups C, G1, G2 and G3 respectively).

### Cytotoxicity

#### Cell viability (MTT) assay results

**Figure 2:**
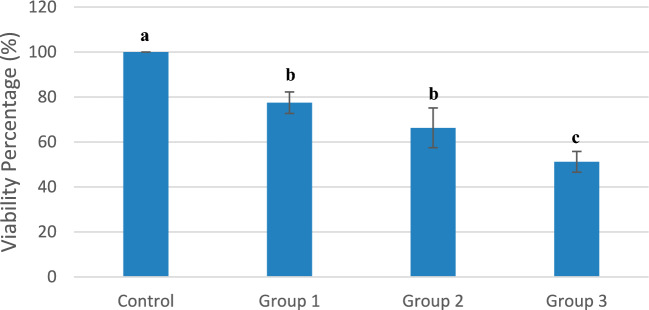
Mean oral epithelial cells viability percentages at 24 h among study groups. Different letters on top of chart columns indicate statistically significant differences at p ≤ 0.05.

The mean value of the viability percentage of the control group was considered to be 100% and the mean viability values of the other groups were calculated according to it. Groups 1, 2, and 3 showed a significant decrease in mean cell viability compared to the control group (Fig. 2). Group 3 expressed the lowest mean viability value (51.19%) and the highest viability among the test groups (1, 2 and 3) was noted in group 1 (77.49 ± 4.78). Post hoc analyses showed significant differences between any two groups except between groups 1 and 2.

#### Cytological evaluation

**Figure 3:**
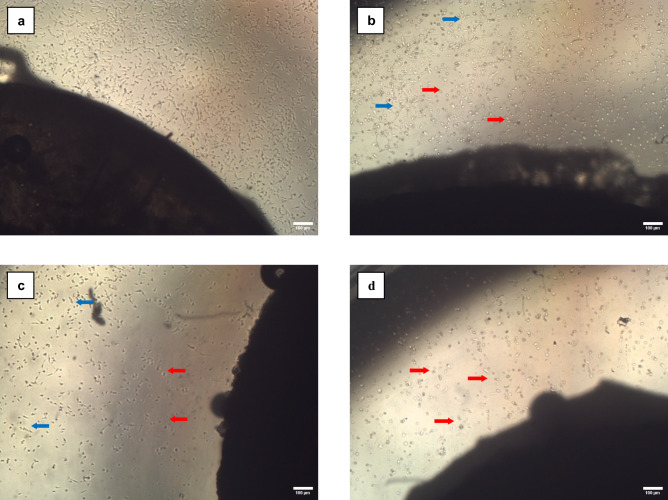
Photomicrograph of the OECs after 24 h of culture on the specimens of the different groups (orig. mag. X10). (**a**) Control group showing cells that were fusiform in shape, with clear and continuous edges; (**b**) Group 1 displaying some fusiform cells (blue arrow), and some cells with a round morphology (red arrow); (**c**) Group 2 exhibited relatively fewer cells than group 1 but with similar morphological patterns; (**d**) Group 3, majority of the cells appeared to exhibit round morphology (red arrow).

Based on the cytological evaluation, it was observed that the cells in the control group exhibited more confluent cells that maintained their morphology as compared to the test groups (groups 1, 2, and 3). On the other hand, the test groups exhibited cells with a more round morphology, compared to the control group. Group 3 seemed to have relatively fewer cells than the other groups and morphologically the majority of these cells appeared to be round (Fig. 3).

On performing Pearson correlation after for the test groups, there was a highly significant positive correlation between the counts of *C. albicans* after different periods (2 days, 4 days, and 6 days) and the percentage of OECs viability. As regards the counts of *S. mutans*, there was only a highly significant positive correlation with the percentage of viability on days 2 and 4 while there was a non-significant correlation on day 6 (p = 0.124) as illustrated in Table 1.

**Table 1:** Correlation coefficient between the percentage of OECs viability after 24 h and the counts of *Candida albicans* and *Streptococcus mutans* percentage.

	Counts percentage of viability_24hr (among study groups 1, 2, 3)	
	r-test	P value
*C. albicans*		
After 2 days	0.896	0.001*(sig)
After 4 days	0.882	0.002*^(sig)^
After 6 days	0.884	0.002*^(sig)^
*S. mutans*		
After 2 days	0.890	0.001*^(sig)^
After 4 days	0.850	0.004*^(sig)^
After 6 days	0.552	0.124^(non-sig)^

### Contact angle

**Figure 4:**
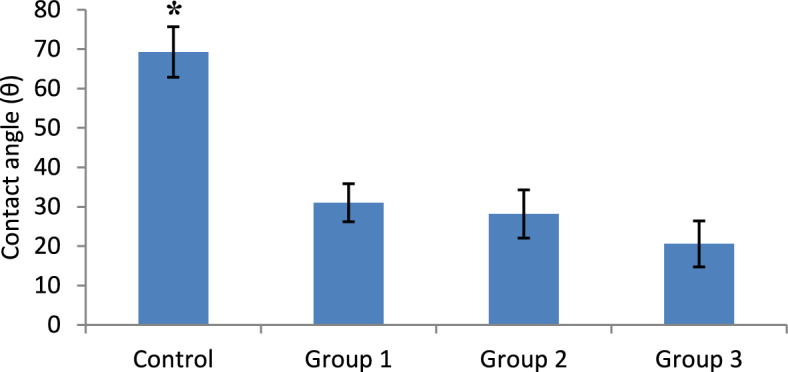
Mean contact angle and standard deviation of wettability among the study groups. *Indicate statistically significant difference between groups at p ≤ 0.05.

Using one-way ANOVA, there was a highly significant difference between study groups (p < 0.001) as shown in Fig. 4. The highest mean value (67.5 degrees) of contact angle was observed among the control group and then decreased gradually among other groups to reach 22.0 degrees in group 3. Post hoc analyses showed significant differences between control and any other group.

### Hardness

**Figure 5:**
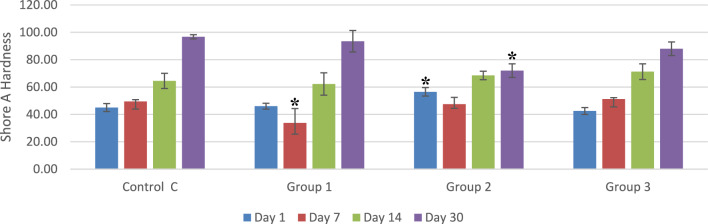
Mean Shore A hardness and standard deviation of the study groups at days 1, 7, 14 and 30. *Indicate statistically significant difference between groups on the same time point at p ≤ 0.05.

Figure 5 illustrates the analysis of hardness at various durations. One-way ANOVA revealed significant differences in mean hardness among the groups, except on day 14. On day 1, post hoc analysis revealed that the mean hardness value of group 2 was significantly higher than the mean values of the other groups. On day 7, post hoc analysis showed that group 1 had significantly lower hardness values than the other groups. On day 30, the mean hardness value among group 2 was significantly lower than the corresponding values of the other groups.

On comparing the hardness of the same group along the different durations using post hoc tests, it was found that all groups showed a significant increase in hardness between day 1 and day 30 (p = 0.001, 0.001, 0.031 and 0.001 for C, G1, G2 and G3, respectively).

## Discussion

DMADDM has been used in various dental materials, but this study is the first to use it for modifying an acrylic-resin cold-cure soft-liner. Different DMADDM concentrations have been utilized in this study to impart antibacterial and antifungal effects to soft liners. This is likely to have promising implications for the development of effective strategies to combat biofilm-associated infections of soft-liners. Concurrently, each concentration's cytotoxicity to OECs and effect on physical properties was evaluated and compared to the control group. The comparison of test groups to the control group yielded significant differences, thus the null hypothesis was rejected.

Two microorganisms: *C. albicans* and *S. mutans* were the focus of the microbiological evaluation in the present study, which are known to be prevalent in the oral cavity^12,16^. Atop of the local infections such as denture stomatitis that these microorganisms may instigate, recent studies have recognized a relationship between denture biofilm and systemic diseases, particularly in elderly individuals^6,14,17^. These microorganisms are believed to influence each other through cell–cell interactions and via the secretion of extracellular signaling molecules. Studies have demonstrated that metabolic and glucan-dependent synergism between *C. albicans* and *S. mutans* contribute to enhanced pathogenesis^5,6,14^.

In this study, we evaluated the count and biofilm mass of each of these microorganisms independently to validate the best concentration effect of DMADDM and avoid confounding results. Biofilm mass was investigated since, unlike planktonic microorganisms, microorganisms found in biofilms have shown tolerance to antimicrobial strategies and pose a real challenge^16,19^. Prior research has demonstrated that the formation of biofilms by *C. albicans* on dentures has the potential to not only enhance the survival of fungal cells but also exacerbate inflammation possibly by secreting mycelium protein-Hwp1, aspartic protease Sap4/Sap6, and adhesion gene-ALS3/EPA1^14^.

The results of this study revealed that there is a significant reduction in the counts and biofilm biomass of both strains with increased concentration of DMADDM in the studied groups in comparison to the control group. It is noteworthy also that there was a time-dependent increase in counts and biofilm for the control group with a corresponding reduction in the test groups between 2 and 6 days. These findings align with those of Han et al., 2017, who noted that positively charged quaternary amine N+ can attract the negatively charged cell membrane of bacteria, causing cytoplasmic leakage and disruption of the cell membrane^11^. Additionally, it has been reported that DMADDM can impact the fungal plasma membrane, leading to mono- and di-valent cation as well as ATP leakage subsequently reducing the survival of fungal cells^14^. Furthermore, the hydrophobic denture surface may promote the adherence of *C. albicans* and selectively increase the propensity of hyphal forms of *C. albicans* colonization^20^. In contrast, DMADDM comprises a hydrophilic group that modifies the surface of soft-liner after polymerization rendering it more hydrophilic as reported in the wettability test, which probably contributed to the reduction of the biofilm formation (adhesion) and hyphal development^6,12,14^.

It is worth noting that the counts were not always significantly reduced like the biofilm biomass for both species. Groups 1 and 2 did not show a significant reduction in their *C. albicans* counts between days 2 and 4, while a significant reduction was noted between days 2 and 6. However, group 3 showed a significant reduction in the counts between days 2 and 4 which might signify that *C. albicans* is reduced more rapidly with elevated DMADDM concentration. On the other hand, regarding *S. mutans* counts, although group 3 exhibited significantly less *S. mutans* counts than groups 1 and 2 on day 2 and day 4, it showed no significant reduction between days 4 and 6. In addition, group 2 on day 6 had lower counts than group 3 at the same time interval. This may be attributed to the fact that the metabolic activity or acid production ability of bacteria is different under different drug pressure of DMADDM as reported by Zhou et al.^18^.

The current study assessed the impact of varying concentrations of DMADDM on the viability of OECs. OECs were chosen for this study as the soft-liners tested will be in intimate contact with the oral epithelial cells of the lining mucosa, which necessitates a thorough evaluation of their effect on OEC viability. Concurrently, the DMADDM cytotoxicity towards OECs was correlated with its effect on pathogens. A positive correlation between the microbial counts and the OECs viability was noted in all test groups included in the study except at day 6 for *S. mutans* counts, denoting that benefiting from increased antimicrobial activity comes with its toll on the OECs viability. Even though several reports have documented DMADDM biosafety^6,15,17,19^, the results of this study, disclosed a reduction in viability of OECs with increased DMADDM concentration. Nevertheless, it should be noted that the least mean viability did not fall below 50% of OECs, never crossing the IC 50 limit. The cell viability results varied among the tested groups, with cell viability percentages between 50 and 60% for group 3, and between 60 and 80% for groups 1 and 2. In the present study, DMADDM concentrations used for groups 1 and 2 could thus be classified broadly as non-cytotoxic concentrations considering that the cell viability percentage can be regarded as optimal when the average viability value is 70% or above^21,22^. Similarly, Garcia et al. documented that increased DMADDM concentrations had cytotoxic effects on human buccal epithelial cells^19^.

Arnold et al., stated that the potential adverse effects of different QAMs have not undergone thorough regulatory assessment for human and ecological health^23^. In accordance with this statement, during our research, very few publications regarding mechanisms behind DMADDM cytotoxicity were available, to the best of our knowledge. Jiao et al., have demonstrated that QAMs induce oxidative stress^24^. QAMs are believed to trigger cell cycle arrest, decrease membrane potential of mitochondria, and activate apoptotic signals in mammalian epithelial cells^25^. These same mechanisms may be responsible for the significant reduction in viability observed in our study with increasing concentrations of DMADDM and compared to the control group.

The acrylic-resin soft-liners are fabricated from polymers with low glass transition temperature, and low modulus with the addition of plasticizers to keep them in the soft state at mouth temperature^9^. This helps the polymer to achieve its intended use as a cushion under the hard resin of the denture^26^. With usage time, plasticizers leach out and the liner material loses its softness (hardness increases)^27^. This leads to an increase in voids and cracks that in turn increase microorganism’s accumulation and candida colonization ending up in the development of denture stomatitis^28^. In addition, the increase in hardness impacts the purpose of the soft liner to reduce the forces transmitted to underlying tissues^27^.

Investigating the effect of DMADDM addition on the wettability degree of the modified soft-liner results revealed a significant decrease in contact angle by more than 50% after the addition of 10 vol% DMADDM to resin’s monomer in comparison to control and reached a decrease by about 67% after adding 30 vol%. This may be attributed to the hydrophilic group in the DMADDM structure that allows the increase in hydrogen bonding with water molecules^14,29^.

Increasing the wettability of the liner surface is an advantage in easing the wetting of the denture fitting surface and increasing the denture retention to oral soft tissues^8^. Different attempts have been investigated as adding hydrophilic agents^30^ or surface plasma treatment^31^ to increase the wettability of the denture fitting surface for better adaptation and retention. However, having this property from within the structure composition itself alleviates the need for further steps to acquire it.

Regarding the hardness results, our findings revealed an increase in all groups’ hardness in a time-dependent manner with a significant difference between day 1 and day 14 and 30. This goes along with the findings in other investigations, that stated that acrylic soft-liners lose their content of plasticizers and ethanol over time by leaching out through the open polymeric structure^7,26^. However, group 2 had no significant increase in hardness from day 14 to day 30. In addition, group 2 had a significantly lower hardness at day 30 than all other groups followed by group 3. This could be the balance between the plasticizer's release and increased softness due to the increased water content of the material related to its possible increased water sorption influenced by the elevated hydrophilicity^32^. Mirizadeh et al., reported the use of DMAEMA in hard acrylic denture resin and stated that it increased the water sorption and water content of the resin^33^. DMAEMA has the same negative group contained in DMADDM and it is a part of its structure.

Within the limitations, this study focused on conferring an antibacterial property to the acrylic-based soft-liner with the investigation of the material’s wettability and hardness changes that may influence the microorganisms’ adhesion. From the obtained results, group 2 (6.6% DMADDM total mass fraction) can be considered the group with the concentration that compromises the effects of the DMADDM on the different properties tested in the current investigation. This conclusion is drawn from the results of the oral epithelial cell viability and the hardness test. Group 2 gave moderate cell viability compared to the other two investigated concentrations and it also gave the lowest hardness by day 30. On the other hand, although group 2 concentration didn’t give the best contact angle, still it showed a high reduction in the contact angle (contact angle (θ) = 28.2°) compared to the control group (contact angle (θ) = 69.3°) which is expected to improve denture retention in the oral cavity.

Further studies are needed to investigate the effect of DMADDM addition on the material’s mechanical properties and its adhesion to the acrylic hard resin. In addition, more insights on the resin/DMADDM interaction in the state of cold-cure are required to be revealed. Also, the efficacy of this new material against dual-species biofilms as they enhance each other’s pathogenesis.

## Materials and methods

### Synthesis of DMADDM

DMADDM can be prepared through a Menschutkin reaction. It involves mainly the reaction of a primary, secondary, or tertiary amine with an alkyl or aryl halide^29^. In the present study, 10 mmol of 2-(dimethylamino) ethyl methacrylate (DMAEMA) (lot# BCBF8391V—Sigma-Aldrich) was mixed with 10 mmol of 1-bromododecane (BDD) (lot# BCCD3837—Sigma-Aldrich) and 3 g of ethanol (3.8 ml) through stirring in a small covered baker on a magnetic stirrer at 70 °C for 24 h^14^. Ethanol was then removed through evaporation and a clear liquid was obtained.

#### Acrylic-resin cold-cure soft-liner modification:

**Figure 6:**
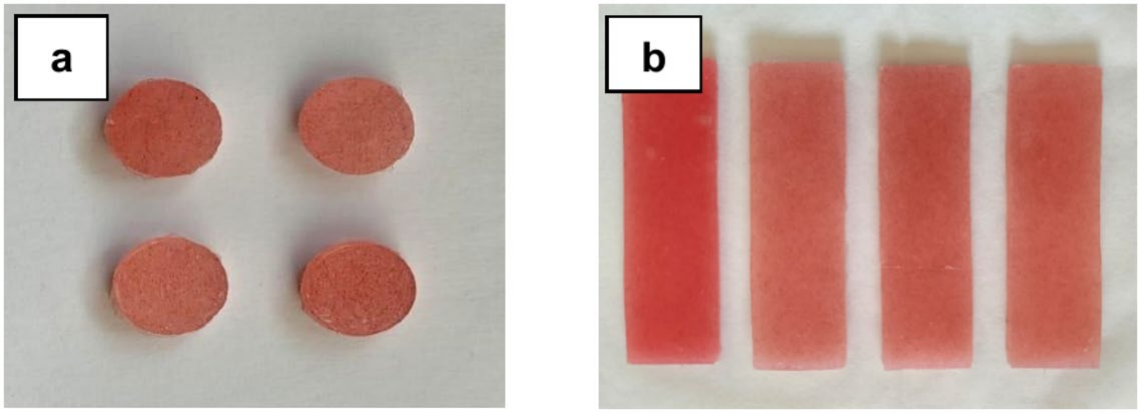
Photomicrograph of study specimens: (**a**) specimens used for microbiological and cytological evaluation; (**b**) specimens used for the wettability and hardness evaluation.

The prepared DMADDM was added to the liquid monomer of the cold-cure soft-liner (Acrostone, Egypt) at different volume percentages (0%, 10%, 20%, 30%) (Table 2) and stirred for 10 min at room temperature. The powder was then added incrementally to the liquid mixture while manually stirring the paste following a ratio of 2:1 (powder:liquid). The obtained paste was put in a Teflon mold and compressed by 1 kg weight. After 15 min, the molds were opened, and the excess material was removed. The molds were then closed again under compression for 6 h to allow the material to fully cure. Disk shape specimens of 1 cm diameter and 0.4 cm thickness were prepared for the microbiological and cytological evaluation, while rectangular shape specimens of 5 × 2 × 0.4 cm (length × width × thickness) dimensions were used for the wettability and hardness evaluations (Fig. 6).

**Table 2:** Study groups descriptions.

Group name	Volume percentage in liquid monomer (%)	Total mass fraction (%)
Control	0	0
Group 1	10	3.3
Group 2	20	6.6
Group 3	30	10

### Microbiological evaluation

#### Microorganism strains and the cultivation conditions

*Streptococcus mutans* (ATCC UA159; Manassas, VA, USA) and *Candida albicans* (ATCC 10231; Manassas, VA, USA) were used in this study for testing the biofilm formation and its quantification by colony forming unit (CFU) count. For *S. mutans,* the bacterial stock was maintained in Brain Heart Infusion (BHI) broth supplemented with 20% glycerol at – 80 °C. Before tests, *S. mutans* were cultured for 48 h at 37 °C with 5% CO_2_ on BHI agar using the prepared glycerol stock. In 5 ml of BHI broth, overnight cultures were created, and they were statically cultivated for 16 h at 37 °C with 5% CO_2_^34^. While *C. albicans*, its stock was kept in Sabouraud Dextrose Broth (SDB) supplemented with 20% glycerol at – 80 °C. Before starting the assay, *C. albicans* were plated from the glycerol stock on Sabouraud Dextrose Agar (SDA) and incubated for 18 h at 37 °C and in 5 ml broth, overnight cultures were prepared^35^.

#### Microorganism biofilm growth

In this test, three disk-shaped specimens (n = 3) of 1 cm diameter and 0.5 cm thickness from each group were used to assess the biofilm formation using the *S. mutans* and *C. albicans.* Each specimen was inserted inside the wells of a 12-well polystyrene tissue culture plate and then sterilized for 30 min under an ultraviolet light source. The overnight cultures for both microorganisms were normalized to an optical density of 1.5 × 10^8^ CFU for *S. mutans*^34^ and 10^6^ CFU for *C. albicans*^36^. Each well contained 500 µl of either the normalized culture of *S. mutans* or *C. albicans* and incubated for 2, 4, and 6 days. After incubation, the supernatants were decanted carefully and were washed once with 1 ml of sterile PBS before subsequent analysis^37^.

#### Evaluation of the colony count for microorganism biofilm

Using sterile forceps, the specimens were removed and transferred into a 15 ml falcon tube, to get rid of non-adherent cells, and washed three times with PBS. Subsequently, 1 ml of PBS was added, and the tube was gently shaken^34,37^. One ml of each sample was transferred aseptically to test tubes containing sterile PBS to reach different dilutions from 10^–1^ to 10^–5^^38^. Every suspension was vortexed for 60 s following each transfer. From each dilution, 100 µl were pipetted and plated on BHI agar or SDA and were spread using a sterile bent glass rod. BHI agar plates were incubated for 48 h at 37 °C with 5% CO_2_ while, SDA plates were incubated at 37 °C from 48 h to 7 days^39^. After incubations, *S. mutans* and *C. albicans* CFU/ml were counted.

#### Biofilm staining with crystal violet

Biofilms were transferred to microcentrifuge tubes. After transfer, biofilms were stained with 1 ml of 0.5% crystal violet for 30 min, washed three times with distilled water, and eluted with 1 ml of 33% acetic acid for 10 min. The solubilized crystal violet solution (100 µl/well in triplicates) was transferred to a 96-well plate, and the absorbance was measured at 570 nm. Average absorbance values were used to quantify biofilm formation for each specimen^34,37^.

### Cytological investigation

#### Cell line and culture

Human Oral Epithelial Cell line (OEC) was obtained from the Medical Experimental Research Center (MERC), Faculty of Medicine, Mansoura University, Egypt. The cells were maintained in Dulbecco's Modified Eagle Medium F12 (DMEM F12) (Sigma, Germany) supplemented with 10% FBS (Sigma, Germany), Penicillin 10,000 Units/ml, Streptomycin 10 mg/ml, Amphotericin-B 25 μg/ml (Lonza, USA) in a humidified atmosphere of 5% CO_2_ at 37 °C.

Specimens were ultraviolet (UV) irradiated by placement in a UV decontamination device (Biological Safety Cabinet-Bioair-07943) for 3 h while flipping them every 30 min on different sides. Specimens were then placed in a 1% (v/v) antibiotic antimycotic solution (10,000 U/ml penicillin G, 10 mg/ml streptomycin sulfate, and 25 mg/ml amphotericin-B diluted in sterile phosphate-buffered saline (PBS) for 24 h at 4 °C. Following sterilization, the scaffolds were washed five times with sterile PBS on a roller for 15 min each time**.**

As cells reached 80% confluency, OECs were cultured on 3 sterilized specimens (n = 3) from each group at a density of 50,000 cells/well in a 24-well plate. Each group was viewed using the inverted light microscope (Olympus IX70, Tokyo, Japan) and the morphological features were noted.

#### Cell viability (MTT) assay

After 24 h, the viability of the cells for all groups was evaluated using the 3-(4,5-dimethylthiazol-2-yl)-2,5-diphenyltetrazolium bromide (MTT) assay. The formazan crystals that were generated were solubilized in Dimethyl sulfoxide (DMSO), and absorbance at 570 nm (A570) was subsequently compared to that of the control. The viability of OECs on each modified-specimen was quantified as the cell viability percentage (%) with the formula:

$$\text{Cells Viability Percentage} \left(\%\right)=\left(\frac{\text{A570 from modified specimens}}{\text{A570 from control}}\right)\times 100$$

### Contact angle (wettability testing)

The surface wettability degree of the control and modified soft-liner specimens was determined by static water contact angle measured by Drop Shape Analyzer (DSA25B, KRÜSS GmbH Co., Germany) using the sessile drop method at room temperature (25 ± 1 °C). Briefly, a drop of deionized water (1 µl) was placed on the specimen's flat surface using a motor-driven micro-syringe mounted vertically against the specimen. As the water droplet was applied on the surface, the droplet arc and the angle of contact (θ) at the interface were recorded by a charge-coupled device (CCD) camera. At least six different records at different sites on the surface of each specimen (n = 3) were obtained and the average values for contact angles were calculated based on the recorded images of the water drops.

### Hardness

The hardness of the specimens was tested according to ASTM D2240 (Standard test method for rubber property—Durometer hardness). Shore A durometer (Model 306L, PTC instrument, USA) was used to measure the hardness of three specimens from each group (n = 3) with a thickness of at least 6 mm. Hardness was measured at 5 different locations 6 mm apart on each single specimen with respect to being away from margins at least 12 mm.

### Statistical methods

The sample size was calculated for each study parameter using Power Sample software NCSS PASS–version 11.0. As the study included four groups, we used one-way ANOVA test to calculate the sample size required. Data entry was done using the Microsoft Excel program then data sheets were exported to IBM-SPSS version 21 for statistical analyses. Quantitative variables were summarized using the mean and standard deviation (SD). The data was explored for normality by checking the data distribution using the Kolmogorov–Smirnov and Shapiro–Wilk tests. Comparison between study groups was done using analysis of variance test (ANOVA). When there was a significant difference obtained, from the ANOVA test, post hoc analyses (pairwise analyses) were applied using the Tukey test. The Pearson correlation coefficient test was used to examine the correlation between the results of the microbial counts and oral epithelial cells viability. The significance level was set at p-value < 0.05.

### Ethics approval and consent to participate

Ethical approval was obtained from Future University in Egypt, faculty of oral and dental medicine under no. FUE.REC (45)/12-2023.

## Conclusion

This study concludes that DMADDM addition to acrylic-resin cold-cure soft-liner is effective in imparting antimicrobial properties against both bacterial and fungal species with possible moderation of hardness increase over time. In addition, it improves the liner wettability which may enhance the prosthesis retention in the patient’s mouth while decreasing some fungal species adhesion. However, balancing these benefits with cytotoxicity is crucial. Higher DMADDM concentrations exhibited increased cytotoxicity to oral epithelial cells, necessitating careful selection of the optimal concentration for safe and effective clinical use.

## Data Availability

The datasets used and/or analyzed during the current study are available from the corresponding author upon reasonable request.
